# TLR9 and COVID-19: A Multidisciplinary Theory of a Multifaceted Therapeutic Target

**DOI:** 10.3389/fphar.2020.601685

**Published:** 2021-01-15

**Authors:** Gillina F. G. Bezemer, Johan Garssen

**Affiliations:** ^1^ Utrecht Institute of Pharmaceutical Sciences, Faculty of Science, Utrecht University, Utrecht, Netherlands; ^2^ Impact Station, Hilversum, Netherlands; ^3^ Department of Immunology, Nutricia Research BV, Utrecht, Netherlands

**Keywords:** pathophysiology, immunology, biomarker, drug target identification, mitochondrial DNA, toll-like receptor 9, severe acute respiratory syndrome coronavirus 2, coronavirus disease 2019

## Abstract

By mapping the clinical pathophysiology of the novel coronavirus disease 2019 (COVID-19) against insights from virology, immunology, genomics, epidemiology and pharmacology, it is here proposed that the pathogen recognition receptor called toll like receptor 9 (TLR9) might have a pivotal role in the pathogenesis of COVID-19. Severe Acute Respiratory Syndrome Coronavirus 2, is causing the greatest global social and economic disruption since world war II. Lack of a vaccine, lack of successful treatment and limitations of the healthcare workforce and resources needed to safeguard patients with severe COVID-19 on the edge of life, demands radical preventive measures. It is urgently needed to identify biomarkers and drug candidates so that vulnerable individuals can be recognized early and severe multi-organ complications can be prevented or dampened. The TLR9 COVID-19 hypothesis describes a mechanism of action that could explain a wide spectrum of manifestations observed in patients with severe COVID-19. The introduced hypothesis proposes biomarkers for identification of vulnerable individuals and positions TLR9 as a promising multifaceted intervention target for prevention and/or treatment of COVID-19. TLR9 agonists might have value as prophylactic vaccine adjuvants and therapeutic immune stimulators at the early onset of disease. Additionally, in this current manuscript it is proposed for the first time that TLR9 could be considered as a target of “inhibition” aimed to dampen hyperinflammation and thrombotic complications in vulnerable patients that are at risk of developing late stages of COVID-19. The readily availability of TLR9 modulating drug candidates that have reached clinical testing for other disorders could favor a fast track development scenario, an important advantage under the current high unmet medical need circumstances regarding COVID-19.

## Introduction

### COVID-19 Unmet Need

The COVID-19 (Coronavirus disease 2019) pandemic, caused by the novel Severe Acute Respiratory Syndrome Coronavirus 2 (SARS-CoV-2) has been declared a public health emergency of international concern by the WHO Director General (WHO, January 29, 2020). The virus, first identified in Wuhan City, China, has spread worldwide, resulting in more than 65M confirmed cases and over 1,5M cases (COVID19.who.int, December 6, 2020). At the time of this writing there are no validated specific therapies with proven effectiveness available for prevention of mortality from COVID-19. Remdesivir has been shown superior to placebo in shortening the time to recovery in adults who were hospitalized with Covid-19 but no significant benefit on mortality could be found (Beigel et al., 2020). Remdesivir is approved in certain countries for treatment of severe COVID-19, while awaiting further evidence and supply. Poor treatment options and the exceptional high burden of COVID-19 on healthcare systems still demands radical preventive measures including travel restrictions, social distancing and lockdowns, resulting in the most severe global social and economic disruption since world war II (Gossling et al., 2020; Dhama et al., 2020). Time-lines to bring a safe and efficacious vaccine for SARS-CoV-2 to market has been proposed to take 12–18 months under ideal circumstances (Billington et al., 2020). Even if intense collaboration and resource allocation can speed up vaccine development it remains a challenge to get the product to the most vulnerable individuals in time. With daily rising new cases and next waves of infections ongoing, it is urgently needed to identify and validate biomarkers and drug candidates so that vulnerable individuals can be recognized early and severe multi-organ complications can be prevented or dampened. This will help to reduce mortality rates and minimize the high pressure on the limited intensive care capacity and healthcare workforce (Adams and Walls, 2020; Dhama et al., 2020; Rolim Neto et al., 2020; Rabaan et al., 2020). Drug candidates and cell-based therapies for management of COVID-19 are being explored in ongoing clinical trials and results are eagerly awaiting (Lythgoe and Middleton, 2020; Khoury et al., 2020; Sanders et al., 2020; Schijns and Lavelle, 2020). Meanwhile, there are still pieces of the puzzle missing, which presents acute unmet medical needs. Patients with severe COVID-19 display a wide array of complications affecting multiple organs including the lungs, cardiovascular system, muscles, brains, liver and kidneys. Further unraveling the mechanisms underlying severe COVID-19 pathology is essential to uncover biomarkers and therapeutic concepts while making efficient use of resources available to allow rapid development.

### TLR9 COVID-19 Hypothesis

**FIGURE 1:**
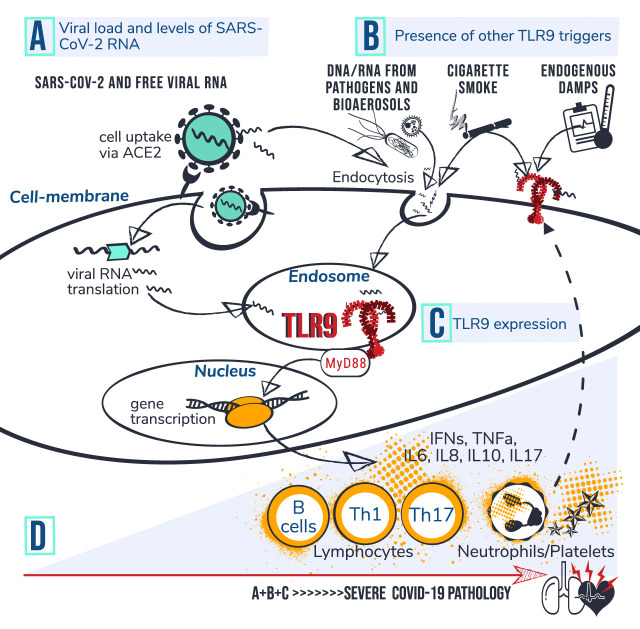
TLR9-Covid-19 hypothesis. Set of circumstances suggested to drive COVID-19 poor outcome via TLR9 encompass; **(A)** viral load and levels of viral RNA; **(B)** presence of other TLR9 triggers, and; **(C)** TLR9 expression levels. **(D)** Individuals with high accumulated levels of A, B and C are proposed to be at risk for developing severe COVID-19 pathology. It is suggested that CpG motifs from SARS-CoV-2 reach TLR9 via ACE mediated viral uptake in the cell followed by RNA translation and transfer of viral CpG-motifs to the endosome. Circulating CpG motifs from virus and other sources could reach TLR9 via endocytosis or directly bind to cell surface at an inflamed site. Dashed line indicates that activation of platelets and neutrophils can increase TLR9 expression levels at cell surface which is suggested to drive a vicious circle of inflammation. Activated TLR9 induces downstream cascades via MyD88, leading to gene transcription, cytokine production and activation of lymphocytes, neutrophils and platelets. The Uncontrolled prolonged activation of TLR9 is suggested to contribute to severe COVID-19 pathophysiology.

**FIGURE 2:**
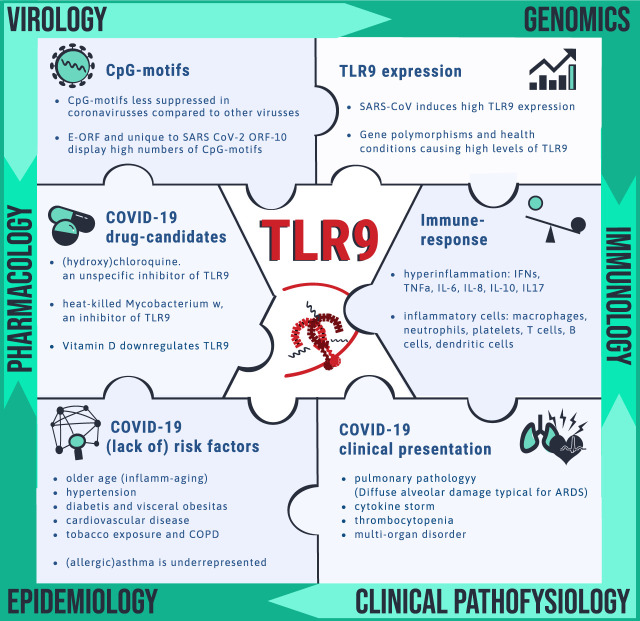
Clues pointing toward drug target TLR9 for COVID-19. Unravelling the mechanism by which SARS-CoV-2 is causing disease is needed for identification of vulnerable patients and for drug target identification. Pieces of the complex puzzle are being filled in by insight from various disciplines including virology, genomics, immunology, clinical pathophysiology, epidemiology and pharmacology. It is proposed that TLR9 could fill in a blank spot worthwhile for further investigation. The bullet points summarize the wide spectrum of observations that can be explained via the TLR9 COVID-19 hypothesis.

Toll-like receptors (TLRs) are a family of 13 conserved transmembrane receptors that are at the forefront of directing innate and adaptive immune responses against invading bacteria, fungi, viruses and parasites (Akira, 2003; Takeda and Akira, 2004; Pasare and Medzhitov, 2005). When TLRs recognize structurally conserved pathogen-associated molecular patterns (PAMPs) they recruit intracytoplasmic TIR domains and specific adaptors such as MyD88, TIRAP and TRIF to control intracellular signaling pathways leading to the synthesis and secretion of appropriate cytokines and chemokines by cells of the immune system (Takeda and Akira, 2004). Among the TLR family, TLR3, TLR7, TLR8 and TLR9 are predominantly localized in intracellular compartments and form the key gatekeepers in detecting and combating viral infections (Akira and Hemmi, 2003). TLR3 is activated by viral double stranded RNA (dsRNA), whereas TLR7 and 8 recognize viral single stranded RNA (ssRNA) and bacterial RNA. TLR9 recognizes RNA and DNA motifs that are rich in unmethylated Cytosine-phosphate-Guanine (CpG) sequences. CpG-motifs are higher expressed in the bacterial and viral genome compared to the vertebrate genome (Hemmi et al., 2000). TLRs can also be activated by endogenous damage-associated molecular patterns (DAMPs) which is believed to have a function in both immune system alert and tissue homeostasis (Bianchi, 2007; Kono and Rock, 2008). Human mitochondrial DNA (mtDNA), evolutionary derived from endosymbiont bacteria, contains unmethylated CpG-motifs and is an example of a well-known DAMP that triggers inflammatory responses directly via TLR9 during injury and/or infection (Zhang et al., 2010). In the setting of COVID-19, multiple TLRs are likely relevant in viral combat and investigations of TLRs as therapeutic target are starting to emerge. Control of the cytokine storm by means of immunomodulators, including TLR7 and TLR8 antagonists and inhibitors of cellular mediators downstream of TLRs such as recombinant human IL-6 monoclonal antibody have been proposed and are currently under clinical investigation (Ye et al., 2020; Felsenstein et al., 2020; Lythgoe and Middleton, 2020; Poulas et al., 2020; Patra et al., 2020). Moreover, the TLR7 agonist, Imiquimod, is proposed as candidate to manage early stage COVID-19 patients (Angelopoulou et al., 2020). The effectiveness of TLR9 agonists for the use as vaccine adjuvants has also been suggested (Oberemok et al., 2020).In contrast to the available papers that more broadly focus on TLR3, 7 and 8, the here presented work, elaborates specifically on the role of TLR9 in defense against SARS-CoV-2 and introduces the hypothetical positioning of exaggerated TLR9 activation in severe COVID-19 pathology. The hypothesis is in line with our previously proposed synergistic disease driving effect of TLR9 agonists in the setting of COPD (Bezemer et al., 2012). TLR9 is broadly expressed on different cell types including epithelial cells in the lungs and nasal mucosa, in muscles and brains, on plasmacytoid dendritic cells and B cells, monocytes, macrophages, neutrophils, megakaryocytes and platelets, T lymphocytes, and NK cells (Hornung et al., 2002; Hayashi et al., 2003; Cognasse et al., 2005; Roda et al., 2005; Fransson et al., 2007; Kabelitz, 2007) A link between TLR9 activation and disease progression in COVID-19 is not directly obvious, since clinical investigations regarding safety and efficacy of inhaled TLR9 agonists in humans reported normal vital signs and no serious adverse effects although some “subtle” effects including moderate nature of flue like adverse events such as chills, fatigue, headache, myalgia and fever have been shown but are considered acceptable (Jackson et al., 2018). On the other hand, TLR9 activation in the airways in mice using high dose CpG-motifs, does lead to inflammation in the airways, ARDS, and sepsis (Knuefermann et al., 2007; Schwartz et al., 1997). Moreover, genetic mutations leading to TLR9 gain of function in human is associated with immune-mediated disease and with a higher incidence of ICU acquired infection (Chatzi et al., 2018; Ng et al., 2010). The TLR9 COVID-19 hypothesis proposes that in specific vulnerable patients, activation of TLR9 could be a silent but driving force explaining the worsening of hyperinflammation and thrombotic complications caused by SARS-CoV-2. Positioning TLR9 in COVID-19 pathology, could explain multi-organ complications and aligns with the fact that only a relatively small proportion of patients infected with SARS-CoV-2 develop severe symptoms requiring ICU. Figure 1 depicts a set of circumstances and a mechanism of action of the proposed contribution of TLR9 to severe COVID-19 pathology in vulnerable patients. It should be noted that the TLR9 COVID-19 hypothesis does not rule out relevance of other TLRs in COVID-19 but rather highlights that disease caused by SARS-CoV-2, could have a worse outcome in people that are A) less well equipped to clear the virus, B) have to deal with a lot of available TLR9 stimuli over a longer period of time and, C) have high expression of functionally active TLR9. This hypothesis is relevant because it can be translated into a multifaceted window of opportunity for existing TLR9 modulating drug candidates that, depending on the disease stage, initially could stimulate, but later on preferably inhibit the TLR9 pathway in vulnerable patients. Moreover TLR9 expression levels and presence of TLR9 ligands are measurable and could potentially provide biomarkers for better identification of a group of individuals at risk for developing a more severe outcome of SARS-CoV-2 infection. High TLR9 expression levels can result from either genetic predisposition, people are simply born with it, or TLR9 expression is upregulated due to underlying health conditions, which will be explained further in the next sections. Examples of synergistically acting triggers for TLR9 include CpG-motifs from co-infecting pathogens, inhaled bioaerosols and organic dust, and cigarette smoke (Bezemer et al., 2012; Bauer et al., 2013; Martinez-Colon et al., 2019; Sun and Metzger, 2019). On top of the previously mentioned mtDNA, released from damaged host cells, also altered self-ligands, called carboxy-alkyl-pyrrole protein adducts (CAPs), that are generated during oxidative stress, are known to aggravate TLR9/MyD88 pathway activation (Zhang et al., 2010; Panigrahi et al., 2013). CAPs have been shown to promote platelet activation, granule secretion, and aggregation *in vitro* and thrombosis *in vivo* (Panigrahi et al., 2013). It is interesting to note that circulating mtDNA levels increase with age which is a familiar trait contributing to chronic inflammation, so called “inflamm-aging” in elderly people (Pinti et al., 2014). This TLR9 axis of inflamm-aging could have relevance in the context of COVID-19 where older age is associated with greater risk of development of severe complications of COVID-19. Figure 2 provides a summarizing overview of insight from different disciplines that reason the hypothesis that TLR9 specifically could have a key role in disease caused by SARS-CoV-2. Further clarification is provided in the next sections.

## Multidisciplinary Clues that Reason the Proposed Role for TLR9 in COVID-19

### Virology: Presence of TLR9—Activating CpG-Motifs

In 2004, TLR9 has been linked to SARS coronavirus induced disease because of the relatively high numbers of CpG motifs in corona viral sequences (Ng et al., 2004). A paper by Ng et al., showed that human coronavirus 229E and Avian infectious bronchitis virus both contain 3 copy numbers of the CpG specific signaling motif GTCGTT, SARS-CoV viral sequence contains 7 copies number while other viruses involved in respiratory diseases have zero CpG motif copy numbers (Human rhinovirus B, Human parainfluenza virus 1, Human respiratory syncytial virus and human metapneumovirus) (Ng et al., 2004). Suppression of CpG motifs is a known mechanism of many mammalian RNA viruses, including influenza virus for adaptation to human host (Greenbaum et al., 2008). Evolving CpG suppression can help the virus to escape from the Zinc Finger Antiviral Protein (ZAP), which is a host antiviral factor that selectively binds to CG-dinucleotide-enriched RNA sequences to degrade target viral RNA (Luo et al., 2020; Gao et al., 2002; Takata et al., 2017). In the context of SARS-CoV-2, ZAP, expressed in human lung cells, has been identified as an important antiviral effector of the IFN response needed to combat SARS-CoV-2 (Nchioua et al., 2020). The authors showed that knock-down of ZAP significantly increased SARS-CoV-2 production in lung cells. The overall CpG composition of SARS-CoV-2 is lower than for other members of the betacoronavirus genus (Xia, 2020) but SARS-CoV-2 does present specific CpG “hotspots” in genomically disparate regions (Digard et al., 2020). The study of Digard et al., showed an over-representation of CpG-motifs within the Envelope (E) open reading frame (E-ORF) and ORF10 of SARS-CoV-2 which is well conserved across the sequences obtained from bat, pangolin and human (Digard et al., 2020). Of the 4 major structural proteins of coronaviruses, the enigmatic E protein, is the smallest protein, involved in several aspects of the virus’ life cycle, such as assembly, budding and envelope formation has also been implicated in the pathogenesis of coronaviruses (Schoeman and Fielding, 2019; Jimenez-Guardeno et al., 2014). During the replication cycle, E is abundantly expressed inside the infected cell, but only a small portion is incorporated into the virion envelope (Venkatagopalan et al., 2015). Across the Coronaviridae, E genes exhibit remarkably high variation in CpG composition, with those of SARS and SARS-CoV-2 having much higher CpG content than other coronaviruses isolated from humans. Moreover, E-ORF displays CpG suppression in all human-infecting viruses except SARS-CoV and SARS-CoV-2, suggesting a potential correlation between CpG presentation and disease severity in human-infecting coronaviruses (Digard et al., 2020). Notable about ORF10 is that this tiny gene, located toward the end of the viral genome, provides a short unknown protein or peptide that is unique to SARS-CoV-2 and uniformly presented in different geographical regions around the globe, and potentially a key protein responsible for SARS-CoV-2 highly contagious nature (Seema, 2020; Khailany et al., 2020; Koyama et al., 2020). The high number of CpG-motifs present in the nucleotide sequence of E-ORF and ORF10 which is unique and specific to SARS-CoV-2 warrants further investigation of a potential role of TLR9 activation in the highly severe and unique to SARS-CoV-2 disease pathogenesis.

### Immunology: Inflammatory Mediators and Cellular Responses

Via the TLR pathways, including TLR9/MyD88, a plethora of inflammatory mediators and cell types can be triggered such as type 1 IFNs, TNFa, IL-6, IL-8, IL-10, IL-17 and activation of Th1 and Th17 lymphocytes, B cells, dendritic cells, neutrophils and platelets (Hemmi et al., 2000; Bezemer et al., 2012; Mortaz et al., 2010; Schwartz et al., 1997; Knuefermann et al., 2007; Greene et al., 2005; Takeda and Akira, 2005; Tasaka et al., 2009; Panigrahi et al., 2013; Hayashi et al., 2003). All these mediators and cell types have also been identified as potential contributors to the so called cytokine storm and thrombotic complications underlying the multi-organ pathological condition in patients with severe coronavirus infections (Li et al., 2020; Cheung et al., 2005; Channappanavar and Perlman, 2017; Birra et al., 2020; Huang et al., 2020; Tay et al., 2020). A clue pointing specifically toward a role for TLR9 in defense against coronaviruses, arises from a paper published in 2004 describing that in response to SARS-CoV infection, TLR9 on human PBMCs from healthy donors was surprisingly high expressed in comparison to other TLR receptors (*p*-value of 0.016) (Ng et al., 2004). The array data from the authors *in vitro* model system showed monocyte-macrophage cell activation, coagulation pathway upregulation and cytokine production together with lung trafficking chemokines such as IL8 and IL17, which were possibly activated through the TLR9 signaling pathway because of the high TLR9 expression levels and the Coronaviridae specific lack of CpG suppression in distinct regions. The TLR9 COVID-19 hypothesis, further elaborates on the idea that specific health conditions of the host that upregulate TLR9 expression contribute to TLR9 mediated inflammation which could potentially explain the differences in severity of the immune response against SARS-CoV-2 between COVID-19 patients. A pro-inflammatory status of the host for instance can drive susceptibility for TLR9 pathway activation by altering cell specific TLR9 expression levels (McKelvey et al., 2011). Life style factors such as a high fat diet and obesity are known to increase TLR9 expression in visceral adipose tissue (Nishimoto et al., 2016 MAR; Thomalla et al., 2019 FEB). Exposure to cigarette smoke, which is also a risk factor for severe COVID-19, causes increased expression of TLR4 and TLR9 on lung CD8(+) T cells of COPD patients and causes increased cytokine production (Nadigel et al., 2011 NOV 9). Upregulation of TLR expression in response to environmental stimuli has also been demonstrated in neutrophils and platelets. Study by Lindau et al. showed that primary blood neutrophils express functional TLR9 on the cell surface, a pathway that can be triggered when pathogen-derived TLR9 ligands cannot reach the endosome, offering a rescue mechanism for neutrophil activation (Lindau et al., 2013 AUG). Incubation of resting platelets with CpG motifs, showed that platelets, when primed, express TLR9 on their surface prior to signal transduction through TLR9 (Panigrahi et al., 2013).

### Genomics: TLR9 Gain of Function Polymorphisms

There are many examples of genetic predisposition leading to TLR9 gain of function. One example is the single nucleotide polymorphism (SNP) of the C allele of rs5743836 (T-1237C), which is associated with immune-mediated disease and with a higher incidence of ICU acquired infection (Chatzi et al., 2018; Ng et al., 2010). T-1237C creates a loop of TLR9/IL-6 signaling amplification, leading to a deregulation in B-cell activation and proliferation upon CpG stimuli (Carvalho et al., 2011 NOV 23). Interestingly TLR9-1237T/C polymorphism is a risk factor for progression of infection to severe sepsis in patients with a male sex predisposition, which was investigated in a pediatric intensive care unit (p 0.014) (Elsherif et al., 2019). Also the SNP rs187084 (T-1486C) of the TLR9 promoter previously being associated with rheumatic disease (Hegazy et al., 2019), cancers and pulmonary tuberculosis (Bharthi et al., 2014) has been suggested to provide relevant risk estimates for the development of sepsis and multiple organ dysfunction in critically ill patients (Chen et al., 2011). A study performed among workers in swine operations furthermore showed that male workers, with polymorphisms of rs187084 in the TLR9 gene, displayed significantly lower lung function than those with wild-type (Gao et al., 2018). Sex differences in TLR9 expression has also been reported in mice, where male mice showed higher expression of TLR9 and higher activation of innate immune system with higher numbers of infiltrating neutrophils upon MCMV viral infection but similar viral load between male and female (Traub et al., 2012). Research performed in HIV patients furthermore showed that TLR9 stimulation by viral CpG DNA contributes to HIV immunopathogenesis and the TLR9 polymorphisms 1635A/G and 1486C/T being associated with disease progression (Joshi et al., 2019). Differences in adverse outcome of Covid-19 between ethnic groups may also in part result from genetic predisposition. Recently Yuval Tal *et al.* analyzed immune factors influencing racial disparity in Covid-19 mortality rates, which revealed presence of inherent differences in the immune system, which may increase the predisposition of black Americans to a severe cytokine storm (Tal et al., 2020). The authors detected elevated expression of markers of innate immunity, including TLR7 and TLR9, and concluded therefor that black individuals would be more prone to develop a rapid and more aggressive cytokine storm.

### COVID-19 Clinical Pathophysiology

#### Pulmonary Pathology

The airways as principal site of entry and target of SARS-CoV-2 can become severely affected in patients with COVID-19. In vulnerable patients, COVID-19 leads to the development of severe pneumonia with enhanced neutrophilia and complications including ARDS requiring mechanical ventilation (Guan et al., 2020). Postmortem examination of COVID-19 patients reveals diffuse alveolar damage with severe capillary congestion and variegated findings in lungs (Menter et al., 2020; Chen et al., 2020). Patients with preexisting lung diseases, including COPD and current smokers might be at greater risk of developing severe complications from Covid-19 (Alqahtani et al., 2020). A role for TLR9 activation in non-allergic neutrophilic airway inflammation and airway disease including COPD has been proposed previously (Greene et al., 2005; Mortaz et al., 2009; Mortaz et al., 2010; Knuefermann et al., 2007; Schwartz et al., 1997; Tasaka et al., 2009; Faust et al., 2020). Moreover, there is evidence that TLR9 can contribute to the development and worsening of ARDS and ALI (Tasaka et al., 2009; Faust et al., 2020; Huang et al., 2020). A study performed in 224 critically ill trauma patients showed that high levels of the TLR9 activator, mtDNA, are associated with ARDS and mortality which is stronger in patients with polymorphisms associated with increased expression of TLR9 (Faust et al., 2020). The prognostic value of plasma mtDNA in ARDS has also been shown in a single-center observational study in China, where higher plasma mtDNA levels at day 7 after admission indicated poor outcome of ARDS patients (Huang et al., 2020). In the airways, however the exact role of TLR9 in disease remains controversial (Bezemer et al., 2012). There is also mounting evidence for a protective role of TLR9 activation in the case of allergic asthma and rhinitis (Iwamura and Nakayama, 2008; Kline and Krieg, 2008; Gupta and Agrawal, 2010). This aligns with the interesting finding that, against odds, asthmatics, seems to be underrepresented among patients suffering from severe COVID-19 of which the current understanding is still in its early stages (Liu et al., 2020). Medication use such as inhaled corticosteroids (ICS) could potentially modify the risk of developing COVID-19 or the clinical course of COVID-19, but at present time there is no robust evidence of such conclusion (Demircan et al., 2000; Celebioglu, 2020; Maes et al., 2020). Reduced expression of ACE2 and transmembrane protease serine 2 (TMPRS2) resulting from ICS use is a potential explanation that has been put forward for understanding the individual difference in susceptibility of severe disease outcome from COVID-19 between asthma patients (Demircan et al., 2000). By other groups of researchers the question arises whether asthma is actually protective against COVID-19 and “work in progress” suggests that a Th2-skewed immunity may be protective against severe COVID-19 disease (Carli et al., 2020). Allergic asthma is a lung disease with a typical Th2 mediated eosinic inflammation whereas COVID-19 presents low level of eosinophils and it is even reported that blood eosinophils decrease during SARS-CoV-2 infections (Lu and Wang, 2020; Sun et al., 2020 Aug). Based on the TLR9 COVID-19 hypothesis, it is proposed that TLR9 mediated combat against COVID-19, as an accompanying effect could result in the sequestration of eosinophils. There is a large body of work showing that TLR9 agonists reduce eosinophilic inflammation and this approach has reached phase 2 clinical testing in human (Iwamura and Nakayama, 2008; Kline and Krieg, 2008; Gupta and Agrawal, 2010). CpG-ODNs effectiveness in the control of allergic responses can be explained by the TLR9 induced T helper 1 (Th1) response that in turn can prevent or reprogram the typical allergic Th2 polarization of the immune system (Chu et al., 1997; Krieg, 2002; Krieg, 2002; Kline et al., 2002). In this context TLR9 has been shown to induce regulatory T cells (Tregs) as well which could potentially contribute to beneficial immunosuppression in allergic asthmatic patients (Ehrlich et al., 2017; Moseman et al., 2004; Kim et al., 2016;), but also provide immune escape opportunity for SARS-CoV-2. Recent data presented by Grifoni et al. show a predominant representation of a classic Th1 response to SARS-CoV-2 with little to no Th2 cytokines (Grifoni et al., 2020).

#### Thrombotic Complications

Evidence is accumulating for a correlation between severe outcome of SARS-CoV-2 infection and abnormal thrombotic complications, vascular damage, dangerous blood clots, and stroke, (Tang et al., 2020; Arachchillage and Laffan, 2020; Guan et al., 2020; Menter et al., 2020; Oudkerk et al., 2020; Spiezia et al., 2020; Wang et al., 2020a; Zhou et al., 2020). COVID-19 ARDS patients compared to non-COVID-19 ARDS patients develop significantly more thrombotic complications mainly pulmonary embolisms with significantly different coagulation parameters (Helms et al., 2020). Thrombocytopenia, decreased blood platelet count, at early stage of disease is associated with poor prognosis in COVID-19 patients (Zhao et al., 2020, Yang et al., 2020). The lung-specific entry of SARS-CoV-2 could drive platelets to the lungs as one of the first lines of defense and also explains the presence of megakaryocytes in the lungs of COVID-19 patients (Thachil 2020; Lefrancais et al., 2017; Salamanna, 2020). Platelet activation can occur via multiple signaling pathways of which platelet-TLR9 has been positioned as a connector between oxidative stress, infection and platelet activation (Panigrahi et al., 2013).Of all TLRs, TLR9 is most highly expressed on platelets as analyzed in the Framingham Heart Study sample population (n = 1625) (Koupenova et al., 2015). Moreover this study showed that a high mean BMI, which is also a major risk factor for COVID-19, is consistently associated with higher TLR expression on platelets. A statistically significant (*p* < 0.05) association with cardiovascular disease measure and TLR9 gene expression was observed in patients that receive lipid treatment (Koupenova et al., 2015). TLR9 can shift the balance of a key initiator of coagulation, called tissue factor and tissue factor pathway inhibitor toward the procoagulant phenotype in human coronary artery endothelial cells and activated blood coagulation in mice (El Kebir et al., 2015). Also functional TLR9 signaling in neutrophils is a mechanism in early stasis experimental venous thrombogenesis (El- Sayed et al., 2016). Neutrophil extracellular traps (NETs) are part of the innate immune response to infections, can form a scaffold and stimulus for platelet adhesion and thrombus formation (Fuchs et al., 2010). NETs have been proposed to contribute to organ damage and mortality in COVID-19 (Barnes et al., 2020). mtDNA is a potent inducer of NETs that activates PMN via TLR9 and formation of mtDNA-induced NETs can completely be blocked by a TLR9 antagonist (Itagaki et al., 2015).

#### Multi-Organ Dysfunction

**FIGURE 3:**
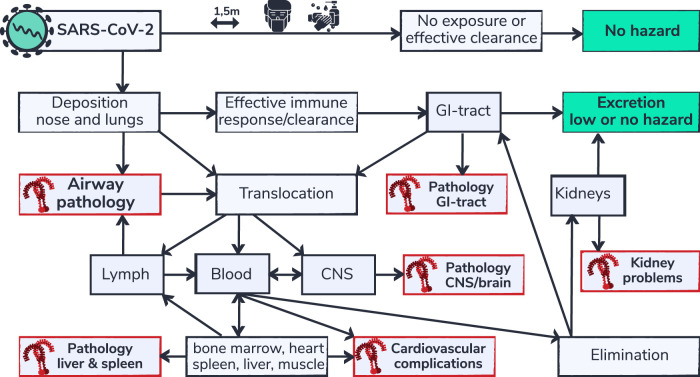
SARS-CoV-2 respiratory deposition and multi-organ complications. Health effects of inhaled substances, including inhaled viruses are influenced by the effectiveness of clearance capability and the routes of elimination. Depicted is a flow chart (adapted from Bezemer, 2009) of body compartments that can be reached via the airways. SARS-CoV-2 is not hazardous for people that are able to avoid exposure or that are able to effectively eliminate the virus from their system. However people that are not able to eliminate the virus or that are vulnerable may develop complications. Organs for which TLR9 mediated pathology is described in literature in non-COVID-19 settings are indicated in red. Regional build-up of SARS-CoV-2 and/or viral RNA, due to inefficient clearance capability in those organs, is proposed to contribute to the typical multi-organ pathology in patients susceptible for TLR9 pathway activation.

Besides lung pathology and thrombotic complications, post mortum case-series show COVID-19-related pathological changes in various organs including liver, kidney, spleen, muscles and brain (Tabary et al., 2020). SARS-CoV-2 can reach from brain to toes and uncertainty over whether it is the virus itself or the response by a person’s immune system makes it hard for doctors to decide on appropriate treatment (Ledford, 2020). The hazard of inhaled substances is influenced by regional deposition sites within the respiratory tract; the effectiveness of the hosts clearance capability and translocation routes to other organs (Bezemer, 2009). The airways as primary site of SARS-CoV-2 infection, facilitates the virus and viral residue components to translocate to multiple organs within the body, which could in part explain the multi-organ complications that are seen in COVID-19 patients (figure 3). Translocation of intact SARS-CoV-2 to other body compartments could give rise to localized increase of viral load because ACE2, identified as key point of entrance of SARS-CoV-2 into the host cell, is widely expressed in tissues including oral and nasal mucosa, nasopharynx, lung, stomach, small intestine, colon, skin, lymph nodes, thymus, bone marrow, spleen, liver, kidney, and brain (Hamming et al., 2004). High expression of ACE2 in the human olfactory epithelium relative to upper airway epithelial cells may explain why COVID-19 is associated with loss of smell and suggest a potential entry point of SARS-CoV-2 into the central nervous system causing neurological symptoms in COVID-19 patients (Chen et al., 2020; Mao et al., 2020). The potential contribution of the nose-brain-barrier and blood-brain-barrier, to brain pathology caused by inhaled hazardous compounds has been described previously (Bezemer, 2009; Oberdörster and Utell, 2002; Tjalve et al., 1996). Dating back 1941, Bodian and Howe showed that a virus is able to move along the axons of neurons (Bodian and Howe, 1941).When they instilled the virus of poliomyelitis in the nose of monkeys, paralytic poliomyelitis resulted only when the olfactory connections were intact. Bovine herpesvirus 5 infection, associated with fatal neurological disease in cattle, invades the CNS mainly via the olfactory pathway and has been associated with overexpression of TLR3, 7 and 9. Mann et al. found a significant increase in the expression of TLRs 3 and 7–9 in the anterior cerebral cortex during acute infection and viral reactivation. In the trigeminal ganglia, only TLR9 expression was significantly affected (Mann et al., 2014). Butchi et al. show that TLRs have differing effects in modulating viral pathogenesis and in direct toxicity in the central nervous system (Butchi et al., 2011). They show that intracerebroventricular inoculation of a TLR9 stimulant induces a more robust neuroinflammation with higher levels of proinflammatory cytokines and chemokines produced by plexus cells that did stimulation of TLR7. The TLR9 mediated increase in cytokines and chemokines correlated with breakdown of the blood-cerebrospinal fluid barrier and recruitment of peripheral cells to the CNS(Butchi et al., 2011). Based on the TLR9-COVID-19 hypothesis it is speculated that if SARS-CoV-2 and/or viral RNA could indeed translocate and accumulate in the CNS it may provoke localized immune responses via TLR9 potentially controllable via TLR9 immune modulators. TLRs, owing presence and having an immune-regulatory role within the brain are identified as attractive therapeutic target for numerous CNS disorders and infectious diseases (Hanke and Kielian, 2011). Similar to the high TLR9 expression in the brain, TLR9 is also highly expressed in skeletal muscle tissue (Nishimura and Naito, 2005). Based on the TLR9 COVID-19 hypothesis it is proposed that TLR9 could also play a role in the observed muscle weakness in COVID-19 patients. TLRs, including TLR9 also play an important role in many if not all types of renal inflammation (Anders et al., 2004). TLR9 via expression on renal infiltrating antigen presenting cells during immune injury have been reported to be involved in antigen-induced immune complex glomerulonephritis, renal vasculitis and lupus nephritis (Anders et al., 2004). Studies performed in experimental models for polymicrobial sepsis show that circulating mtDNA via activation of TLR9, contributes to cytokine production, kidney injury during and splenic apoptosis (Tsuji et al., 2016). Other experimental studies furthermore show that TLR9 is an important mediator of hepatic injury secondary to ischemic acute kidney injury (Bakker et al., 2015). Inhibition of TLR9 in mice attenuates sepsis induced mortality and provides dampening of dysregulated inflammatory markers in spleen, lung and liver (Hu et al., 2015).

### Epidemiology: Riskfactors/Comorbidities Associated with Overweight and Obesity

Early epidemiological data revealed that SARS-CoV-2 is more likely to affect older males with comorbidities, and can result in severe and even fatal respiratory diseases such as ARDS and multiple organ failure (Chen et al., 2020). Reported comorbidities in infected patients that require hospital admission include cardiovascular disease/heart disease, diabetis mellitus, chronic respiratory disease, hypertension and cancer (Butchi et al., 2011; Arumugam et al.,; Huang et al., 2020; Wu and McGoogan, 2020 APR 7). Obesity, has been positioned as common denominator of impaired metabolic health, respiratory dysfunction, cardiovascular disease and diabetes mellitus in the severe course of COVID-19 (Stefan et al., 2020). Preliminary investigations show that people with obesity are at increased risk of severe COVID-19 (Goyal et al., 2020; Halasz et al., 2020; Stefan et al., 2020). The exact mechanisms through which obesity exacerbates COVID-19 infection are not fully clarified. The association of obesity with immune and metabolic derangement is one explaining suggestion for the link to adverse clinical outcomes in COVID-19 (Korakas et al., 2020). Studies in mice show that obesity induced by high fat diet or leptin deficiency result in overexpression of TLRs and related proinflammatory signaling molecules in enlarged adipose tissues, which may play an important role in the obesity-associated phenomenon of meta-inflammation (Kim et al., 2012). A high fat diet increases TLR9 expression in visceral adipose tissue in mice (Nishimoto et al., 2016 MAR). TLR9 expression is also significantly increased in visceral compared to subcutaneous adipose tissue depots in obese patients (Thomalla et al., 2019). The function of TLR9 in adipose tissue inflammation remains controversial. On the one hand it has been suggested that TLR9 may protect against obesity and the metabolic syndrome having an anti-inflammatory effect (Hong et al., 2015; Thomalla et al., 2019). On the other hand it has also been shown that obesity induced single stranded DNA (ssDNA), released from adipocytes stimulate chronic adipose tissue inflammation and insulin resistance via TLR9 (Nishimoto et al., 2016 MAR). Additionally the study from Nishimoto showed that plasma concentration of ssDNA was significantly higher in patients with visceral obesity compared to patients without visceral obesity and ssDNA was positively correlated with visceral fat area (Nishimoto et al., 2016 MAR). Ghosh et al. proposed a role for TLR9 in the activation of plasmacytoid dendritic cell fueling obesity induced chronic low-grade inflammation, so called meta-inflammation (Ghosh et al., 2016). Revelo et al. provided data on TLR9 pathway involvement in promoting obesity related inflammation of metabolic tissues including visceral adipose tissue and liver. In mice a high fat (HFD) diet induces excess of nucleic acids and related protein antigens which worsens metabolic inflammation through activation of VAT macrophages and expansion of plasmacytoid dendritic cells (pDCs) in the liver (Revelo et al., 2016). The study of Revelo furthermore confirmed that HFD-fed mice lacking TLR9, show reduced metabolic inflammation and treatment of HFD-fed mice with a TLR7/9 antagonist improved metabolic disease. A more recent study from Yuzefovych et al., showed that plasma mtDNA is elevated in obese type 2 diabetes mellitus patients and is associated with oxidative stress in skeletal muscle and correlates with insulin resistance (Yuzefovych et al., 2019). TLR9 message and protein expression levels which are higher in diabetic wounds compared to control wounds have been linked to impaired wound healing in type 2 diabetes mellitus (T2DM) cases via the induction of pro-inflammatory S100A8 and IL-8 (Singh et al, 2016). The *TLR9-1237 T*/*C* gene polymorphism is considered as a molecular risk for diabetic foot among patients with T2DM (Wifi et al., 2017).

### Investigational Treatment Approaches of COVID-19

#### Chloroquine and Hydroxychloroquine

Chloroquine and Hydroxychloroquine are medications approved for prevention and treatment of malaria with a reputation of being effective and relatively safe for treatment of systemic lupus erythematosus and mild to moderate rheumatoid arthritis because of immune suppressive properties (Rainsford et al., 2015). Chloroquine is a well-known, however not specific, inhibitor of endosomal TLRs, including TLR9 (Kuznik et al., 2011). Chloroquine and Hydroxychloroquine have been shown to inhibit SARS-CoV-2 *in vitro* and it is speculated to be effective for patients with COVID-19, although until now no single study shows any validated and proven clinical benefit (Sanders et al., 2020; Wang et al., 2020b). Also, the exact mechanism by which (Hydroxy)Chloroquine is believed to relief infection by a coronavirus remains unclear. Suggestions for (Hydroxy)Chloroquine mechanism of action include alteration of the acidic environment inside lysosomes and late endosomes, preventing endocytosis, exosome release and phagolysosomal fusion, and inhibition of the host cytokine storm (Tripathy et al., 2020). Concerns exist about using off-Label drugs for COVID-19 including Chloroquine and Hydroxychloroquine, because of the recognized side effects: QT prolongation, torsades de pointes, hepatitis, acute pancreatitis, neutropenia, anaphylaxis and increased risk of cardiac death (Kalil, 2020). Applying reverse thinking moving back from bedside to bench, it could be speculated that the TLR route, including TLR9, could have contributed to reducing overstimulation of the immune-system in the individual COVID-19 patients that experienced benefit from investigational off-label treatment with (hydroxy)chloroquine. In experimental models, TLR9 signaling is recognized as a major target for the protective actions of Chloroquine in the case of sepsis induced acute kidney injury (Yasuda et al., 2008). From this viewpoint, The specific blocking the TLR9 pathway in vulnerable critically ill COVID-19 patients, might even be a more targeted approach with potentially less side effects than investigational broad-spectrum (hydroxy)chloroquine. But keep in mind that at this point TLR9 modulation is not a treatment recommendation since more (pre)clinical research is needed to investigate the proposed hypothesis.

#### *Mycobacterium* w

Early clinical findings pointing toward a role for TLRs including TLR9 in COVID-19 disease pathology arise from a study performed with heat-killed *Mycobacterium* w (Mw) (Sehgal et al., 2020). Mw is a cost-effective immunomodulator approved in India for treatment of leprosy, and is investigated for use as vaccine and treatment option for tuberculosis and for use in autoimmune conditions such as psoriasis and optic neuritis (Sudhalkar et al., 2012). Mw received attention in drug discovery for having both TLR2 and 4 activating as well as TLR inhibiting properties, including inhibition of TLR9 (Belani et al., 2011; Sudhalkar et al., 2012; Anwar et al., 2019). A small scale study in which 4 severely ill COVID-19 patients were treated with heat-killed *Mycobacterium* w (Mw), resulted in successful management, not causing adverse events (Sehgal et al., 2015). A previously performed randomized trial in fifty patients with severe sepsis, showed that the use of Mw was associated with significant reduction in days on mechanical ventilation, ICU and hospital length of stay, lower incidence of nosocomial infection, and delta SOFA score (sequential organ failure assessment) (Sehgal et al., 2015). A randomized clinical trial to further evaluate the safety and efficacy of Mw in critically ill patients suffering from COVID-19 is currently ongoing (clinicaltrials.gov: NCT04347174). The exact mechanism by which Mw acts in sepsis remains unknown. In addition to the previously reported TLR antagonistic capability it is also suggested that Mw could enhance TLR activity, which might overcome the immune paralysis in severe sepsis (Sehgal et al., 2015).

### Vitamin D

During the first wave of Covid-19, low Vitamin D levels have been found in the vulnerable aging population in Spain, Italy and Switzerland which pointed towards the potential of vitamin D in prevention of COVID-19 infection and mortality (Ilie et al., 2020). Vitamin D deficiency has indeed been found to contribute to ARDS and a narrative review on vitamin D shows accumulation of evidence that vitamin D supplementation could reduce risk of COVID-19 infections and deaths (Grant et al., 2020). Vitamin D is known to promote innate immune response against viral infection and a role for TLRs has been proposed in explaining the underlying mechanism. Martinez-Moreno et al showed that innate immune response against the dengue virus (DENV) infection, a public health problem worldwide, can be improved by vitamin D supplementation. Their study showed that an oral supplement of 4000 IU/day of vitamin D3 significantly decreased TLR9 protein levels and the mRNA abundance of TLR3, TLR7, and TLR9 in human. The lower dose of, 1000 IU/day of vitamin D only decreased the TLR9 protein level in human monocte-derived DCs infected with DENV. The finding is especially interesting because TLR9 activation, through mtDNA, contributes to DENV-induced immune activation (Martinez Moreno et al., 2020). A study performed in 2010 also showed that intracellular TLRs are differentially regulated by vitamin D3, with TLR9 being down-regulated by vitamin D3 exposure whereas TLR3 was unaffected (Dickie et al., 2010). The study by Dickie et al showed that vitamin D3 decreased TLR9 expression in monocytes and had a downstream functional effect as these cells subsequently secreted less IL-6 in response to TLR9 challenge.

## Multifaceted Potential of Drug Target TLR9 for COVID-19

**FIGURE 4:**
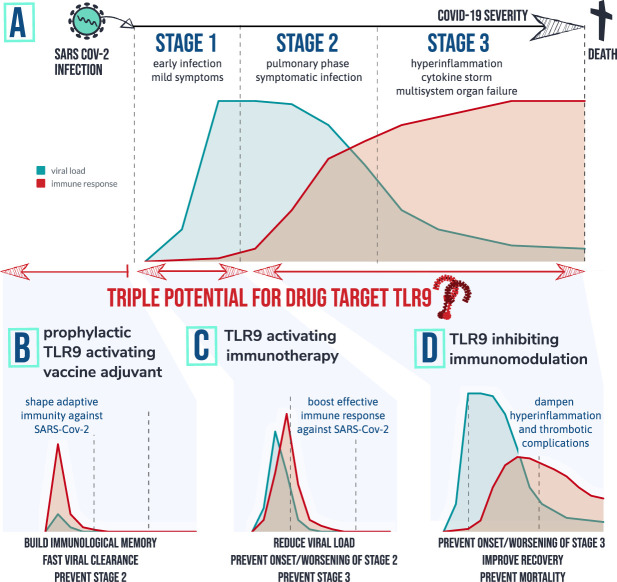
therapeutic implications of the TLR9 COVID-19 hypothesis: Patients that develop severe symptoms of COVID-19 are tend to go through different stages of disease with different characteristics. Graph **(A)** depicts a simplified fictional scenario explaining how an inefficient viral specific immune response at start of infection (stage 1) can result in a high peak of viral load and eventually an exaggerated inflammatory response causing symptomatic infection (stage 2). When the virus remains active and/or the host immune system remains active over prolonged period of time severe complications can occur requiring ICU (stage 3) and in worst case result in death. Based on the TLR9 COVID-19 hypothesis, 3 therapeutic strategies are worthwhile investigating for following desired actions: **(B)** shape adaptive immunity against SARS-Cov-2 so that viral load remains low; **(C)** Provide a short targeted immune boost to help clearing the virus efficiently, and **(D)** Inhibit TLR9 pathway in vulnerable patients to prevent or dampen hyperinflammation and multi-organ complications.

The novel hypothesis that TLR9 could be associated with COVID-19 pathology in vulnerable patients, positions TLR9 as a multifaceted drug target worth considering for preventing and/or treatment of critical conditions of SARS-CoV-2 infected patients. Both TLR9 activation- and inhibition could be relevant to produce opposing therapeutic effects at the different stages of disease (Figure 4). Prophylactic potential of TLR9 activation as vaccine adjuvant to shape adaptive immunity against SARS-Cov-2 is currently being investigated in clinical trials (Oberemok et al., 2020). This would ideally result in immunological memory to aid fast viral clearance thereby preventing severe symptomatic infection and virus induced damage. Also in the early infection stage, prior to complications it could be imagined that activation of TLR pathways including TLR9 could aid in fast and effective viral clearance especially in immunocompromised patients. In COVID-19 it seems that viral burden typically peaks early in illness, potentially even before symptoms of pneumonia and then declines as antibodies develop and antibody titers rise over the subsequent 2 to 3 weeks (Kim et al., 2020; To et al., 2020; Woelfel et al., 2020; Zou et al., 2020). Activation of TLR9 in this early window of disease would ideally result in improved viral combat thereby preventing or shortening of symptomatic infection and prevention of overwhelming viral illness and tissue damaging inflammation. The FDA approved an investigation into the efficacy of an inhalational broad acting TLR2/6/9 agonist, PUL-042 to reduce the severity of COVID-19 in adults positive for SARS-CoV-2 infection (Schijns and Lavelle, 2020). It should be noted that stimulation of other TLRs in this early window of infection could have similar therapeutic value in immunocompromised patients. Imiquimod, for instance is an activator of TLR7 and has been proposed to enhance the innate and adaptive immunity in early stage COVID-19 patients (Angelopoulou et al., 2020). Also other non-viral specific TLRs such as TLR5 which is activated by bacterial Flagellin has been proposed for vaccine or adjuvant development to generate protective innate immunity against SARS-CoV-2 (Chakraborty et al., 2020). In contrast to the numerous potential valuable TLR agonists, it is proposed that TLR9 could be considered as particular interesting target of inhibition because of the lack of CpG suppression in unique to SARS-CoV-2 regions which could be of specific concern in vulnerable patients that experience difficulties to clear the virus and that have more than normal TLR9 expression and/or more than normal synergistically TLR9 triggers present. TLR9 inhibition could thus be a strategy worth considering for treatment of the specific COVID-19 patients that are at risk for developing severe symptomatic infection and further complicated clinical course due to underlying TLR9 skewing vulnerabilities. Risk factors mentioned in this hypothesis paper include (pre)existing thrombotic activation, chronic neutrophilic lung disease, presence of coinfections, high levels of visceral fat, high levels of circulating mtDNA levels, TLR7 loss of function gene polymorphisms and TLR9 gain of function gene polymorphisms. Taken together, the relatively high numbers of CpG-motifs in SARS-CoV2 and the upstream position of TLR9 in the inflammatory cascades and the broad expression of TLR9 on different cell types that play crucial roles in clinical COVID-19 presentation (Th1 cells, Th17 cells, B cells, neutrophils, platelets), TLR9 is positioned be a promising systemic therapeutic target to dampen or perhaps even prevent the thrombotic complications and so called cytokine storm or hyperinflammatory syndrome in certain specific patients that are suffering from severe COVID-19. Dampening of cytokine storm has evident potential for preventing the onset or worsening of ARDS and multisystem organ failure and ideally aid improved and shortened time for recovery, prevention of death and reducing post-ICU complications (Ragab et al., 2020; Ye et al., 2020). For any immunomodulating treatment concept it is however important to determine proper alignment with individual qualitative and quantitative factors of pathogen and host immune interactions. For instance immunosuppressive approaches to reduce hyperinflammation in COVID-19 may lead to unwanted impairment of anti-microbial immunity (Ritchie and Singanayagam, 2020). Moreover TLR inhibition may drive compensatory changes in other TLRs. For instance blocking of TLR7 and TLR8 which is currently being invested in a phase II trial could potentially pose risk to the specific patients that are already skewed toward TLR9 activation. Likewise blocking of TLR9 in patients that do not experience overstimulation of TLR9 may result in loss of an important innate immune signaling pathway that is needed to combat the virus. To prevent risk of viral flare up due toTLR9 antagonistic activity, the antagonist could be tested in combination with Remdesivir and other investigational antivirals. Vice versa excessive activation of a specific immune response for purposes of viral clearance via activation of TLRs, including TLR9 could contribute to hyperinflammation and thrombotic complications in susceptible patients and could therefore be followed up by immunosuppressants in patients that experience complications. This impediment thus asks for a good understanding of individual characteristics that relate to the TLR drug targets.

## PREDICTIVE MARKERS FOR INDIVIDUALS VULNERABLE FOR SEVERE COVID-19

Viral load and viral RNA levels are relevant predictive parameters for disease. Viral load of SARS-CoV-2 detected from the respiratory tract of COVID-19 patients seems positively linked to biochemical indexes and disease severity (Liu et al., 2020). Studies have indicated that the highest viral load in throat swabs can be detected at the time of symptom onset (He et al., 2020). Upon resolution of symptoms, viral RNA levels may remain positive for more than 2 weeks in upper respiratory tract specimens (nasopharyngeal swab and/or an oropharyngeal swab) which is however not necessarily associated with disease severity but may result from a weaker immune response instead (Carmo et al., 2020). The underlying individual factors influencing viral combat capability and viral clearance are likely diverse, therefore challenging to encompass for early predictive purposes. An example of poor viral clearance capability due to a less robust immune response can be found in the association between older age and greater risk of development of ARDS and death from COVID-19 (Wu et al., 2020). Also very specific individual characteristics may contribute to poor viral defense. An example arises from a recent preliminary communication, in which a case series study presented that genetic variants leading to TLR7 loss of function were present in 4 young male COVID-19 patients, all previously healthy with unsuspected severe complications of COVID-19 of which 1 patients died. Besides older age and poor TLR7 function, there could be many more dysfunctional steps in the immune response that could drive high viral load, which goes beyond the scope of this hypothesis paper. Literature covering a more broad perspective of immunological aspects of COVID-19 is available (Felsenstein et al., 2020; Jensen and Thomsen, 2012; Li et al., 2020; Tay et al., 2020; Birra et al., 2020; Ragab et al., 2020). The TLR9 COVID-19 hypothesis proposes that combining measures of viral load and viral RNA with markers for TLR9 susceptibility, would provide a more precise identification of some people at risk, feed into better prevention strategies for those patients and give rationale for more targeted treatment options via modulation of TLR9. In this theory paper we discussed genetic markers including: ZAP, C allele of rs5743836 (T-1237C) in TLR9, -1486 T/C (SNP) rs187084 (T-1486C), 1635A/G and 1486C/T. Mentioned were also life style factors such as high fat diet and cigarette smoke exposure, that can increase TLR9 expression levels. Moreover we discussed the presence of measurable synergistically acting TLR9 triggers originating from other pathogen and from the host. The TLR9 COVID-19 hypothesis proposes to investigate increased levels of mtDNA and ssDN as biomarkers for COVID-19 vulnerability.

### Recommendations

The TLR9 COVID-19 hypothesis is testable within the framework of current knowledge. TLR9 expression levels in response to SARS-CoV-2 can be analyzed in an *in vitro* model system such as used by Ng et al. for investigating genome-wide host response to SARS coronavirus (Ng et al., 2004). Another appropriate approach is to analyze variations in TLR9 expression levels in relevant patient samples such as sputum and/or lung lavage samples from patients with COVID-19 and in affected tissue biopsies from patients that died from severe COVID-19. Animal knockout models could give further insight in the requirement of TLR9 for SARS-Cov-2 induced pulmonary and thrombotic complications, cytokine storm and multi-organ dysfunction. An advantage under the current global emergency circumstances related to COVID-19 is that research groups and pharmaceutical companies showed long lasting interest in immunomodulating agents that engage the TLR9 pathway. There is a large body of preclinical data and early human clinical trial results showing the safety and therapeutic potential of TLR9 modulating compounds to improve vaccines and treat cancer, infectious disease, allergy/asthma, autoimmune disorders (Anwar et al., 2019; Krieg, 2006; Bezemer et al., 2012; Gupta and Cooper, 2008). Prior art that covers safety profiles, dosing, pharmacokinetics, pharmacodynamics could help the repurposing of drug-leads and speed up the drug development process of TLR9 targeting drug candidates for COVID-19. Model systems, including TLR reporter assays and other cell- and tissue-based systems could allow fast screening of available TLR9 modulating lead compounds having the biological effects that are desired in COVID-19 as mentioned in Figure 4. For successful translation from bench to bedside, also a deeper understanding of the spatiotemporal kinetics of viral load and specific host factors is a recommended approach for identification of patients at risk that are most likely to benefit from treatment at defined stages of disease. Conclusions on the relevance of TLR9 as drug target and as predictive marker for identification of people at risk could be drawn from large scale, real world screening of COVID-19 disease severity in relation to the combined measures of A) viral load and SARS-CoV-2 RNA, B) Endogenous and exogenous cell free DNA including mtDNA and ssDNA from visceral fat and DNA from other pathogens, and C) TLR9 polymorphisms and TLR9 expression levels. If the TLR9 COVID-19 hypothesis can be further justified, well-controlled clinical trials to study safety and efficacy of TLR9 modulating drug leads for treatment and/or prevention of disease caused by a coronavirus are warranted. It would also be recommended to evaluate the effect of TLR9 antagonists in combination with Remdesivir or other investigational antivirals on recovery time and mortality rates in adults that are hospitalized with COVID-19.

## Data Availability Statement

The original contributions presented in the study are included in the article/Supplementary Material, further inquiries can be directed to the corresponding author.

## Author Contributions

GB formulated the TLR9 COVID-19 hypothesis, drafted the manuscript and figures, and revised the final form. JG provided key insights for manuscript revision. Both authors contributed to the article and approved the submitted version.

## Conflict of Interest

GB filed a patent entitled “A TLR9 inhibitor for use in treatment of COVID-19,” reference number 1043690.The remaining author declares that there are no commercial or financial relationships that could be construed as a potential conflict of interest to this work.
